# Continuous, real-time monitoring of neonatal position and temperature during Kangaroo Mother Care using a wearable sensor: a techno-feasibility pilot study

**DOI:** 10.1186/s40814-018-0293-5

**Published:** 2018-05-21

**Authors:** Suman Rao, Prashanth Thankachan, Bharadwaj Amrutur, Maryann Washington, Prem K. Mony

**Affiliations:** 1 Department of Neonatology, St John’s Medical College Hospital, St John’s National Academy of Health Sciences, Bangalore, 560034 India; 2 Division of Epidemiology and Population Health, St John’s Research Institute, St John’s National Academy of Health Sciences, Bangalore, 560034 India; 3 Robert Bosch Center for Cyber Physical Systems, Indian Institute of Science, Bangalore, 560012 India; 4 Department of Electrical Communication Engineering, Indian Institute of Science, Bangalore, 560012 India

**Keywords:** Kangaroo mother care, *m*Health, Monitoring, Neonatal health, Temperature, Vital, Signs, Wearable sensor

## Abstract

**Background:**

Remote biomonitoring of vital parameters in hospitals and homes has the potential to improve coverage and quality of maternal and neonatal health. Wearable sensors coupled with modern information and communication technology now offer an opportunity to monitor temperatures and kangaroo mother care (KMC) adherence in a continuous and real-time manner remotely for several days’ duration in hospital and home settings. Using an innovative remote biomonitoring device to measure both temperature and baby position, we undertook a techno-feasibility study in preparation for a clinical trial.

**Methods:**

We designed and developed a wearable sensor for tracking KMC adherence and neonatal temperature, using social innovation design principles. After screening mother-infant dyads using clinical and logistic eligibility criteria, we piloted this wearable sensor along with a gateway device and the commercial cellular network. The dyads were recruited during hospitalization and followed up in the hospital and home phases for several days. Simple descriptive statistical analysis was undertaken.

**Results:**

Recruitment rate was 50% (6/12), and consenting rate was 83% (5/6) during a 2-month period. These five neonates contributed a total of 39 study days (15 hospital days and 24 home days). Their mean [± standard deviation (S.D.)] birth weight was 1490 (± 244) g.

The mean (± S.D.) of the vital signs for the five babies was temperature [36.5 °C (± 0.3)], heart rate [146.5/min (± 14)], and oxygen saturation [94% (± 4)]. No severe or moderate side-effects were noted; one baby developed mild dermatitis under the device that was transient and self-limiting, yielding an incidence proportion of 20% and incidence rate of 2.6/100 person-days.

None of the mothers reported any discomfort with the use of the device. Temperatures detected from 81 paired readings revealed that those from the wearable sensor were 0.2 °C lower than those detected by clinical thermometers [36.4 (± 0.7) *vs* 36.6 (± 0.3); < 0.001].

There was also iterative feedback that was useful for hardware and software design specifications of the wearable sensor, the gateway device, and the analytics platform. Lastly, lessons were learnt with regard to the logistics of research team interactions with healthcare professionals and study participants during the hospitalization and post-discharge home phases of the study.

**Conclusions:**

The pilot study has shown that it is feasible and acceptable to track KMC adherence as well as maternal and newborn temperatures in a potentially safe manner on a real-time mode for several days’ duration during hospitalization and home phases. The pilot has also helped inform modifications in clinical monitoring, technological modifications, and logistics planning in preparation for the definitive clinical trial.

**Trial registration:**

Clinical Trials Registry of India, CTRI/2017/09/009789

## Background

Kangaroo care or kangaroo mother care (KMC), sometimes called skin-to-skin care, is a technique of newborn care where babies are kept skin-to-skin with a parent, typically their mother. It is most commonly used for low birth-weight preterm babies. Since the introduction of kangaroo mother care (KMC) in the 1970s in Colombia, the initial set of studies was designed to show that it was not inferior to incubator care [1, 2]. Subsequently, it has been identified to also contribute to successful breastfeeding, bonding and attachment, better sleep, decreased pain perception during procedures, positive effects on infant development, and increased parent satisfaction. All of these effects have been identified as contributing to reductions in morbidity and mortality among preterm or low birth-weight (LBW)-stabilized infants [3]. However, in the decades since then, it has primarily been seen as an accepted practice mainly applicable for resource-limited settings rather than as a standard of care globally [4].

Earlier studies that elucidated the role of KMC in thermoregulation were limited in their duration of measurement of physiological parameters to a few hours before, during, and after KMC. Further, the field of verification of KMC in actual clinical practice was limited to relying mainly on self-reporting by the mother with no means of validation in hospital or home settings. The newer field of wearable sensors coupled with modern information and communication technology now offer an opportunity to address both these concerns by monitoring temperatures and KMC adherence in a continuous, remote, and real-time manner accurately for several days in hospital and out-of-hospital settings. We designed and developed an innovative remote biomonitoring device that could measure both temperature as well as baby position and then conducted a pilot techno-feasibility study in preparation for a clinical trial of safety and accuracy of the wearable sensor device for measuring temperature and KMC adherence. The objectives of this pilot study were to estimate rates of enrolment, attrition, and side-effects and also to study capture and visualization of kangaroo care and maternal and neonatal temperatures.

## Methods

### Study setting

St John’s Medical College Hospital, Bangalore, is a 1300-bedded, tertiary-care hospital with 2500 deliveries per annum. It has a level 3 nursery and cares for about 1000 neonates (inborn:outborn ratio = 2:1) in the neonatal care intensive unit (NICU) per year, with survival rates of 99% at 48 h. About one third of newborns are low birth weight and one fifth are preterm. Background rates of maternal mortality ratio and infant mortality ratio were 1.33 and 31 per 1000 live births respectively in Karnataka state [5].

### Study design

We employed a techno-feasibility study design to determine the feasibility of the technical aspects, rather than the economic aspects, while setting out to study the safety and potential efficacy of our device [6]. The primary objectives were to assess (i) the acceptability and feasibility of the intervention by measuring rates of neonatal eligibility, consenting, and completion and (ii) the feasibility of visualization of data. Secondary objective was to assess the feasibility of capture of outcome measures (safety and accuracy of device) as measures of efficacy of the definitive trial.

### Prototype and implementation

Two key requirements for the “on-body” sensor were safety and performance accuracy. Given the fragility of the newborn skin, the device had to be hypoallergenic [7] and burst/leak proof [8] and dissipate minimal heat or non-ionizing radiation. The adhesive used to secure the device was to cause minimal “skin injury” [9], allergy, or infections. Device accuracy was targeted to be ± 0.3 °C in in vitro conditions [10] and ± 0.5 °C in actual clinical practice [11]. Other mechanical requirements for the device were long battery life, robustness (without any malfunction on coming into contact with body fluids), dust- and water-proof casing, and human-centric design facilitating continuous use and that the device should not get re-set accidentally. There was also a requirement for the device to store data locally and communicate with a gateway device for onward transmission of data via the commercial cellular network. The wearable sensor enclosures were coin-stack shaped with all the electronics embedded inside.

Based on these requirements, we designed, prototyped, and developed a wearable device, the details of which are given elsewhere [10]. Briefly, the device is 40 mm long, 7 mm thick with a breadth of 32 mm at the broader end, and 27 mm at the narrow end. It weighed 8 g.

**Fig. 1:**
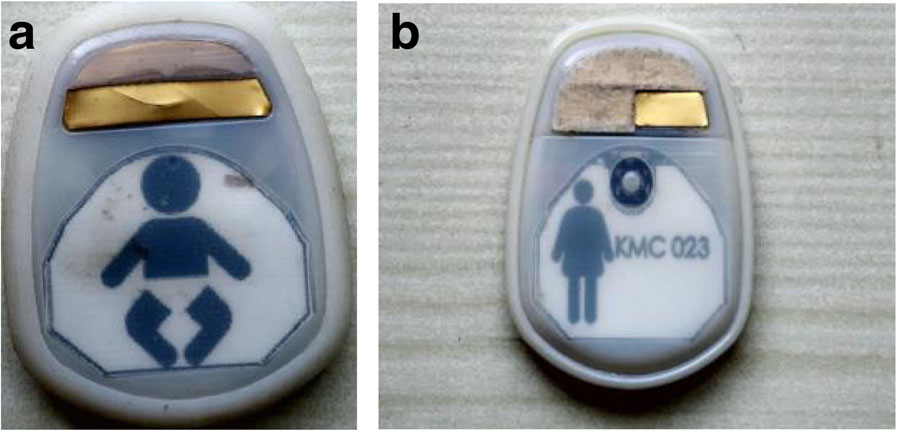
Device with sensors for (**a**) neonate’s side and (**b**) for mother’s side

A baby-friendly enclosure was made from medical-grade hypoallergenic plastics. High-precision thermistors were used for accurate temperature measurements. When the device was sandwiched between the skin surfaces of the mother and the baby, temperatures from both the surfaces were picked up (Fig. 1). Proximity (or touch) sensors allowed for determination of skin contact for both the baby and the mother. A three-axis gyroscope provided three axes of angular rotation with which we could determine if the baby was in the range for optimal/acceptable KMC position (10°–90°). A 3-V coin battery powered the device with a power-saving mechanism of “active” and “sleep” modes. The sensor was programmed to sample readings (temperature as an average of 10 readings over 10 s, angle between 0 and 360, and touch/proximity of mother/baby) once every 6 min. With this sampling rate, the device could function with no battery change for up 3–4 weeks. The sensor then communicated with a gateway device (a smartphone) via very low-energy Bluetooth® (VLBE 4.0) to relay the data over a secured internet backbone provided by GPRS/Wi-fi on to a centralized database for storage.

The pilot study was undertaken during February to March 2016. The inclusion criteria were low birth-weight (LBW) neonates < 2000 g eligible for KMC and on whom kangaroo care had been initiated and continued for at least 2 days prior to start of the study; extreme preterm neonates (corrected gestational age < 28 weeks) were excluded. In addition, the families had to be residing within 2 h of travel from the hospital (for home visits by research assistants) and willing to come back for weekly review clinic follow-up visits after discharge.

One device and cellphone were handed over to each family for the duration of the study along with simple instructions to the parents on correctly securing the device onto the abdomen (as per illustrations shown in Fig. 1) with an adhesive and for checking for wireless connectivity of the phone with the device as well as the cellular network.

### KMC and study measurements

Effective KMC for each baby in our NICU comprised of a combination of “skin-to-skin contact” and “exclusive breastfeeding” or “alternate feeding.” Skin-to-skin contact initiation was after stability criteria were met (respiratory and/or hemodynamically stable, without serious illness, and could tolerate handling). Each episode of KMC was recommended for at least 60 min. The clothing of the kangarooed baby included cap, diaper, and socks. The kangarooed baby was to be prone on the provider’s chest, and the provider was to be reclined at an angle of 30° to 90°.

The remote biomonitoring (RBM) device was positioned over the epigastric region of the neonate’s abdomen. The abdominal skin temperature, despite it being subject to the vasomotor activity of the skin, was deemed to be appropriate for continuous monitoring. In addition to being close to a metabolically active organ (the liver), facilitating a measurement close to the core temperature, it also enabled a non-invasive measurement that was steady, continuous, and easy-to-use. For each recruited newborn, vital signs were monitored for a duration of 1 h per day for up to 5 days in the hospital—the first hour after attachment of the device on day 1 and 1 h per day on the following 4 days. Temperature was recorded every 15 min while heart rate and oxygen saturation were recorded every 30 min. Episodes of KMC were directly observed by a research assistant during hospitalization. After discharge from the hospital, contact with neonate was maintained via a combination of neonatology out-patient review visits once a week, home visits by research nurses once a week, and telephone calls on the interim days.

The feasibility and acceptability of continuous, real-time monitoring of temperature and position were evaluated by measuring recruitment, consent, and study completion rates.

Safety in the short term was also evaluated by the examination of each baby daily for side-effects under the device or adhesive, and if present, were classified as mild, moderate, or severe.

Performance efficacy of the sensor was measured by validating device measurements against routine clinical recordings. Time-stamped abdominal skin temperatures obtained from the device were compared against axillary temperatures measured using a clinical thermometer (mercury or digital reading after 3 min in the hospital and home settings respectively); 0.5 °C was added to these skin temperatures for all analyses, since skin temperatures are usually 0.5 °C lower than axillary temperatures [12]. The position of the baby captured as angle readings or as presence/ absence of “touch” between the device and the skin of baby/mother was compared against reported/ observed KMC episodes. In the hospital, the research nurse annotated the starting and ending times of kangaroo care by direct observation while in the home phase, this was self-reported by the mother. For the hospital phase of testing, direct observation was the “reference standard” against which the device was compared for purposes of validation. In the home phase of testing, the purpose was to see if the device captured the duration of KMC episode reliably. Simple descriptive statistical analysis was undertaken.

### Sample size and data analysis

A sample of 12 newborns was selected based on convenient sampling for this pilot study [13]. Simple descriptive statistics are presented. Data are summarized by mean (± standard deviation) for normally distributed continuous data. For count of events, data are presented as proportions or rates.

### Ethics

Ethics approval for the study was obtained from the St John’s Medical College Institutional Ethics Review Board (IERB # 360/2015 dated 12 Jan 2016). The clinical trial was registered with the Clinical Trials Registry of India (CTRI/2017/09/009789). Informed consent was obtained from parents by research assistant nurses after eligibility of neonate, based on clinical and geographic criteria, was satisfied.

## Results

Out of 12 newborns available during the study period, six (50%) were eligible to take part in the study; three were ineligible because of medical contra-indications and three due to non-medical reasons. Five out of these six families consented to take part in the study. These five neonates completed both the hospital and home phases contributing a total of 39 study days (15 hospital days and 24 home days). The mean (± standard deviation (S.D.)) birth weight was 1490 (± 244) g. The mean (± S.D.) of the vital signs for the five babies was temperature [36.5 °C (± 0.3)], heart rate [146.5/min (± 14)], and oxygen saturation [94% (± 4)].

No severe or moderate side-effects were noted; one baby developed mild dermatitis under the device that was transient and self-limiting, yielding an incidence proportion of 20% and incidence rate of 2.6/100 person-days. None of the mothers reported any discomfort with the use of the device.

Temperatures detected from 81 paired readings revealed that those from the wearable sensor were 0.2 °C lower than those detected by clinical thermometers [36.4 (± 0.7) vs 36.6 (± 0.3); < 0.001]. In the hospital phase, the difference was 0.4 °C [36.1 (± 0.4) vs 36.5 (± 0.4); *p* < 0.001], while in the home phase, the difference was 0.1 °C [6.8 (± 0.7) vs 36.7 (± 0.2); *p* = 0.72].

In the hospital phase, for a total of 13 KMC episodes with direct observation, KMC episode durations picked up by the device were 82% (when angle alone was considered), 69% (when touch was considered), and 61% (when both angle and touch were considered). In the home phase, against a median duration of 3.5 h by self-reporting, the device documented evidence for a median of 1.7 h (by touch), 1.2 (by angle), and 1.0 (by touch and angle combined).

**Fig. 2:**
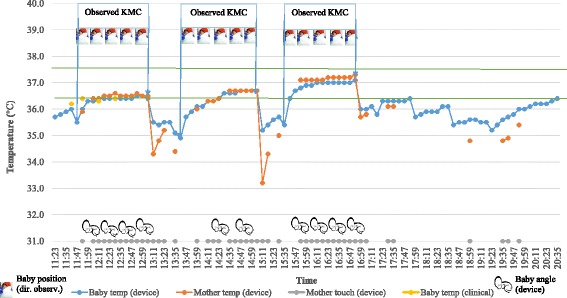
Comparison of wearable device-derived temperatures and KMC recording against routine episodic clinical temperatures and directly observed KMC, respectively, and temperature changes during KMC episodes during hospitalization phase in a low birth-weight baby

**Fig. 3:**
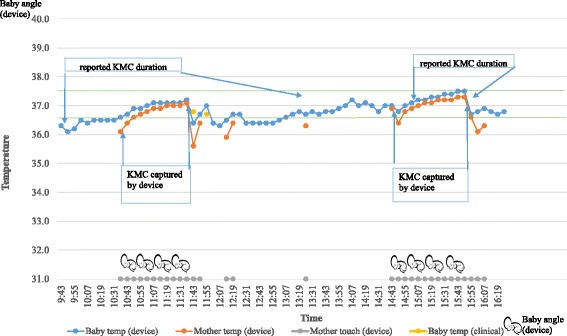
Comparison of self-reported KMC duration against duration capture by wearable device, and temperature changes during KMC episodes during home phase in a low birth-weight baby

**Fig. 4:**
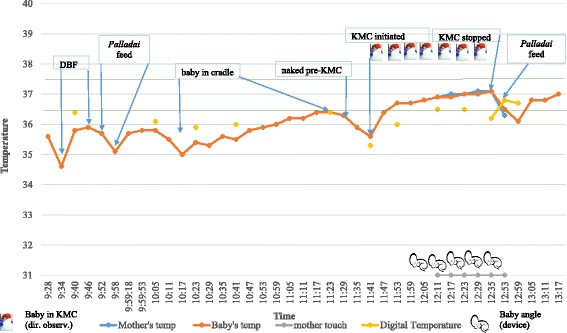
Continuous real-time monitoring of temperature of a preterm neonate during feeding, KMC, and other activities in the hospital

The positive effect of KMC on neonatal thermoregulation can be seen in Figs. 2, 3, and 4. In addition, Fig. 2 also shows device validation of KMC against direct observation in the hospital setting, roughly 90% accuracy of KMC episode duration capture (when angle alone was considered) and 77% accuracy (when touch alone was considered). Figure 3 shows the reliability of self-reporting against the wearable device, that is, 126 min of KMC as recorded by the device and corrected to 140 min (to account for about 10% under-capture by device from Fig. 1) versus 270 min of KMC by self-reporting. Figure 4 illustrates several routine activities in the NICU during KMC and inter-KMC intervals that could affect a newborn’s temperature. These activities include direct breastfeeding (DBF), feeding of baby with expressed breastmilk via a *palladai* (a south Indian traditional infant feeding cup with beak), uncovering baby in preparation for KMC, and episode of KMC.

## Discussion and conclusions

The pilot study has helped address areas of uncertainty prior to starting the future definitive study, a single-arm phase II clinical trial [13]. We were keen to assess feasibility of recruitment while in the NICU, and on-going participation for several days both during the hospitalization phase as well as in the post-discharge phase. We did not know whether families would retain the sensor device by changing the adhesive every day and what type and severity of adverse events might need to be associated with the use of the device and the adhesive in the low birth-weight babies. This study has helped obtain rates of eligibility, consenting, and completion as well as preliminary data on the device safety and performance that helped inform fine-tuning of the study protocol in three different domains—clinical monitoring, technologic implementation, and logistic arrangements—in preparation for the definitive trial.

In clinical monitoring, safety and performance of the device are the two key considerations [14]. Despite apparent physiologic stability during KMC, it is suggested that it is prudent that infants in the NICU have continuous cardiorespiratory monitoring [15]. These parameters were found to be stable during the period of use of the “on-body sensor.” Observed side-effects during the pilot study helped design an elaborate “adverse event reporting and resolution” mechanism [16] with incidence proportion, incidence density, and severity being measures of primary safety outcomes. It also prepared us to get a study dermatologist on board to assist with prompt diagnosis and treatment plans for side-effects that we might encounter during the definitive trial. Accuracy of the device is to be measured as validation of the device against the reference standard of direct observation of KMC episodes and their durations. Precision of abdominal skin temperatures could be fixed − 0.5 °C relative to axillary measurements. For the capture of KMC, angle measurements by the device seemed to be linked closest to direct observation in our pilot study. For reliability testing of the device, it appears that self-reporting by the KMC provider appeared to be over 1.5 times that captured by the device. In addition, with well-documented readings of temperatures linked to KMC episodes in hospital and home settings, we could study the KMC-temperature coherence as well. Our preliminary results are in agreement with earlier studies, such that the better thermal control seen in LBW/preterm neonates could be attributed to skin-to-skin care [2, 17].

Frequency of data capture by the wearable sensor could be programmed to be at intervals of 6 min given that mother’s temperature met each infant’s thermal zone requirements within 5 min of onset of KMC [2]. From a technology viewpoint, this helped in the optimization of battery life. Further, testing of the software application for participant recruitment on cellphones/tablets was feasible and features such as communication with the wearable device, data logging and storage, and data synchronization with the server on the cloud all had “technical bugs” that could be identified and fixed before the start of the main trial.

Illustrations of critical clinical parameters helped inform the creation of a customized dashboard with details of number of devices in the field, number of babies with temperature abnormalities along with graphic visualization of temperatures (neonatal and maternal), and episodes of KMC as defined by touch, angle or touch and angle combined. In addition, it was possible to input data from validation tests such as direct observations and clinical measurements into the database for comprehensive visualization. Further, the software could be programmed to send alerts from the server to the research staff in the form of emails and SMSs regarding system-related information such as the health of the battery (for device and phone). Furthermore, the need to prepare for large volumes of data coming in and planning for intelligent analysis of that data for meaningful interpretation was also appreciated [18].

This pilot study also helped in finalizing preparations for industrial manufacture of devices as well as fine-tuning of logistics arrangements for the hospitalization phase in terms of screening for eligibility, recruitment, and preparation for discharge. For the home phase, it was possible to finalize the frequency of monitoring using a combination of neonatology out-patient review visits once a week, home visits by research nurses once a week, and telephone calls on the interim days. In addition, research nurses also assessed training needs of families with regard to storing, retrieving, or transmitting data from the phones as required.

Adhering to the same inclusion criteria for the main trial as used in this pilot study will likely yield high rates of successful follow-up of study participants. Assuming a device efficacy of 80% accuracy and ±10% relative precision in picking up correct neonatal position and temperature compared to the “reference standards” (being direct observation for neonatal position and axillary readings obtained using clinical thermometers for temperatures), with a two-sided significance of 0.05 and power of 0.8, we anticipate the sample size to be about 100 babies for the definitive trial [13].

Given the growing interest in medical devices globally as well as transitioning from an unregulated market scenario to a more regulated medical device environment in several resource-constrained settings, newer guidelines helped to navigate our pilot study as well [14]. In summary, continuous, real-time monitoring of neonatal temperature and KMC adherence was found to be feasible and acceptable in this pilot study. The study helped inform the clinical, technological, and logistical preparations for the definitive clinical trial of the wearable sensor as part of a future mobile-health architecture.
